# Tuberculosis and immigration: the challenges in the Latin American and Colombian context

**DOI:** 10.1016/j.lana.2023.100600

**Published:** 2023-09-22

**Authors:** Darío Sebastián López, José Fernando Valderrama Vergara, Julián Alfredo Fernández-Niño, Silvia Juliana Trujillo-Cáceres

**Affiliations:** a Faculty of Medicine, Cooperative University of Colombia, Pasto, Nariño, Colombia; b Department of Global Public Health and Bioethics, Julius Center, University Medical Center Utrecht (UMCU), Utrecht, the Netherlands; c Bloomberg School of Public Health, Johns Hopkins University, Baltimore, MD, United States of America; d Department of Global Public Health and Bioethics, Julius Center, University Medical Center Utrecht (UMCU), Utrecht, the Netherlands

Migration patterns in Latin America and the Caribbean are undergoing significant changes, exemplified by the complex situation in Venezuela. Over 7.7 million Venezuelans now live outside their country, with approximately 6.5 million residing in the region.^1^ However, it is estimated that over 9.7 million people will be in need of assistance.^2^ The unprecedented scale of migration flows has led to new challenges for destination countries (as Colombia) in the integration of these populations at different levels, as well as putting strain on limited infrastructure, resources, health, and social services.^3^

These challenges are highest due to the burden of certain communicable diseases that affect populations from low and middle-income countries (LMIC), such as tuberculosis (TB). Although the risk of TB infection and disease in migrants is linked to TB incidence in their country of origin, it is evident that migration processes, particularly those involving forced, irregular migration, of vulnerable individuals, or in contexts of humanitarian emergencies, political, environmental, or economic crises, can increase case transmission.^4^^,^^5^ This situation does not stem from migration itself causing an increase in TB incidence, but rather that the circumstances associated with these types of migration can create conditions, such as overcrowding, poor nutrition, and lack of timely access to health services in host countries, that facilitate the transmission of the disease (Fig. 1).

**Fig. 1:**
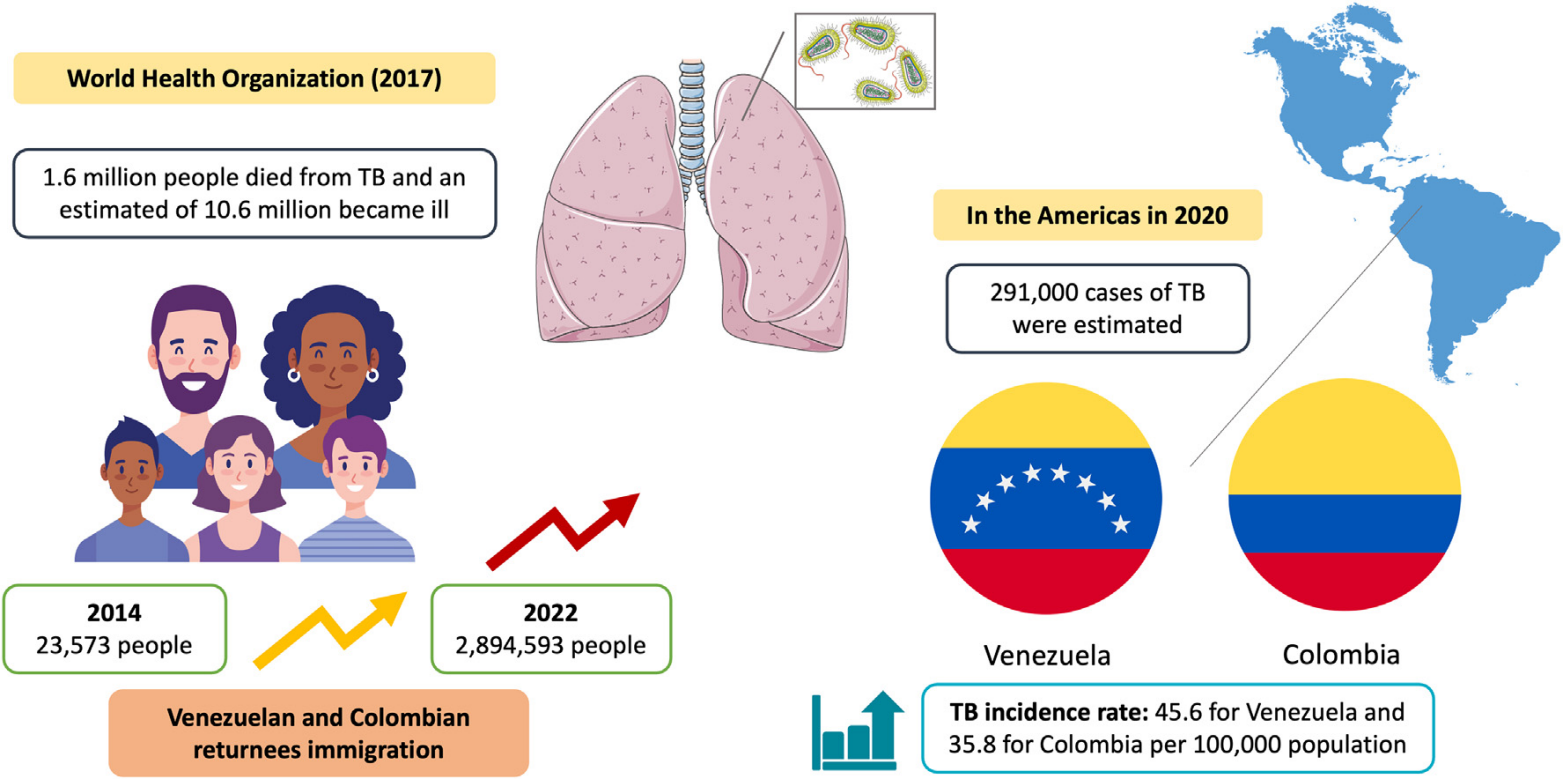
**Migration flows and the Tuberculosis Burden: a focus on Colombia and Venezuela**. Abbreviations: TB, Tuberculosis. Elements of the figures were adapted using icons by Creartive and Amoghdesign from Flaticon, and images from Servier Medical Art, provided by Servier. All are licensed under a Creative Commons Attribution 3.0 Unported License.

**Source**: Bagcchi S, WHO's Global Tuberculosis Report 2022^6^ and OPS, Tuberculosis En Las Américas. Informe Regional 2020^7^

Despite the solidarity and efforts of Colombia to engage access to health services for Colombian returnees and Venezuelan immigrants, providing a regular migration status that would allow access to health insurance and formal jobs in the country, is a high logistical and financial challenge.^8^ As a result of this situation, the increase in public health events of concern, such as TB, requires innovative and sustainable policies and interventions that engage different stakeholders in national and international cooperation actions to respond to this situation in migrant populations.

Greater collaboration is needed to facilitate information sharing and coordinate actions among countries, with a specific focus on including “pendular” migrants and individuals in transit across multiple countries. Ideally, this collaboration should involve not only the transit and destination countries but also the receiving country. In the case of Venezuela, where international political conflicts pose significant challenges, it becomes even more crucial to ensure that public health surveillance and action continue to maintain active channels, transcending these differences.

On top of that, according to the Global TB report, during the COVID-19 pandemic, access to TB care, the burden of TB disease, drug-resistant TB (DR-TB), and HIV coinfection have been a worse impact in the worldwide context, and LMIC.^6^ Further studies are required to quantify the magnitude of this problem in the border regions in destination countries.

In line with the WHO End TB Strategy, it is valuable to empower social civil organizations and communities to intensify interventions on the social determinants of TB in these migrant populations. Also, political commitment is mandatory for strengthening epidemiological surveillance-including community-based surveillance (especially of vulnerable groups) and improving the application and expansion of early diagnosis with new rapid molecular tests. Finally, ensuring access to TB preventive therapy (mainly for contacts under 15 years of age and people living with HIV), and implementing the new oral treatment schemes for DR-TB.^6^^,^^7^

In conclusion, all the above requires an investment of resources and funds to ensure the sustainability of these strategies and interventions in the frame of equal access to healthcare. All stakeholders should strive to join forces and redouble their efforts to achieve the objectives of ending TB. Ending TB in any country or territory is simply impossible without inclusivity for migrants.

## Contributors

DSL: Conceptualization, writing–original draft, investigation; JFVV: writing–review and editing; JAFN: writing–review and editing; SJTC: supervision, investigation, and writing–review–editing.

## Declaration of interests

We declare no competing interests.
